# Involvement of Smac, p53, and caspase pathways in induction of apoptosis by gossypol in human retinoblastoma cells

**Published:** 2012-07-20

**Authors:** Wei-Ting Hsiao, Ming-Dar Tsai, Guey-Mei Jow, Lu-Tai Tien, Yih-Jing Lee

**Affiliations:** 1 School of Medicine, Fu-Jen Catholic University, New Taipei City, Taiwan; 2 Department of Life Science, Fu-Jen Catholic University, New Taipei City, Taiwan; 3 Department of Neurosurgery, Shin Kong Wu Ho-Su Memorial Hospital, Taipei, Taiwan; 4 Graduate Institute of Clinical Medicine, College of Medicine, National Taiwan University, Taipei, Taiwan

## Abstract

**Purpose:**

Retinoblastoma is a malignant tumor of the retina usually occurring in young children. To date, the conventional treatments for retinoblastoma have been enucleation, cryotherapy, external beam radiotherapy, or chemotherapy. Most of these treatments, however, have possible side effects, including blindness, infections, fever, gastrointestinal toxicity, and neurotoxicity. More effective treatments are therefore imperative. Gossypol has been reported as a potential inhibitor of cell proliferation in various types of cancers, such as prostate cancer, breast cancer, leukemia, and lung cancer. This study investigates the possible antiproliferative effect of gossypol on retinoblastoma.

**Methods:**

Human retinoblastoma cells were cultured with various concentrations of gossypol and checked for cell viability with a 3-(4,5-dimethylthiazol-2-yl)-2,5-diphenyltetrazolium bromide (MTT) assay. Nuclear condensation caused by cell apoptosis was detected by staining retinoblastoma cells with 4',6-diamidino-2-phenylindole (DAPI), counting those with condensed nuclei, and determining the percentage of apoptotic cells. In addition, the stages of apoptosis and phases in cell cycles were examined with flow cytometry. The possible signal transduction pathways involved were examined with a protein array assay and western blot analysis.

**Results:**

After incubation, the cell survival rate was significantly lower after treatment with 5, 10, and 20 µM of gossypol. The maximum antisurvival effect of gossypol was observed at 20 µM, and the number of apoptotic cells was higher in the preparations cultured with 10 and 20 µM of gossypol. The results in flow cytometry indicated that at concentrations of 10 and 20 µM, gossypol increased the proportion of early- and late-apoptotic retinoblastoma cells and induced cell arrest of retinoblastoma cells at the same concentrations. This antiproliferative effect was later confirmed by upregulating the expression of death receptor 5 (DR5), caspase 8, caspase 9, caspase 3, cytochrome C, tumor protein 53 (p53), and second mitochondria-derived activator of caspases (Smac) in the signal transduction pathways.

**Conclusions:**

We concluded that gossypol has an antiproliferative effect on retinoblastoma cells.

## Introduction

Retinoblastoma is the most common intraocular malignant tumor in infants and children, where it might occur unilaterally or bilaterally. Most cases of unilateral retinoblastoma involve somatic nonhereditary retinoblastoma 1 gene (*RB1*) mutations, which account for 60% of all retinoblastoma cases [1]. Approximately 40% of retinoblastoma patients carrying a germline mutant of *RB1* have a bilateral retinoblastoma tumor [1], and these patients are more likely to develop secondary tumors. The morbidity of retinoblastoma is approximately 1 per 20,000 to 1 per 15,000 live births in the United States [2], and approximately 10 new cases are reported each year in Taiwan [3]. Retinoblastoma is usually recognized in patients younger than 5 years by leukocoria, strabismus, and pain [1,4]. To date, retinoblastoma has conventionally been treated with enucleation, cryotherapy, external beam radiotherapy, or chemotherapy, depending on the condition and stage of tumor development and the location and size of the primary tumor [5,6]. However, most of these treatments have possible side effects, including blindness, infections, fever, gastrointestinal toxicity, and neurotoxicity. Therefore, more effective treatments are imperative for improving patients’ prognoses. Increasing the rate of complete cure from this disease has long been a priority in ophthalmologic and pediatric clinics.

Gossypol is a polyphenolic extract of cottonseeds. The biologic effects of gossypol are attributable to 6 hydroxyl and 2 aldehyde groups, which also make the molecule soluble in organic solvents. To date, gossypol has had a wide range of uses. In agricultural applications, it reduces larval weight and the metabolic rate in invertebrate pest species by inhibiting mitochondria ATPase activity [7]. Gossypol has also been suggested as a male contraceptive that reduces energy production in spermatozoa, and therefore, their motility and concentration, without affecting testosterone levels [8]. In curative medicine, the anticancer properties of gossypol have been investigated extensively. In studies on prostate cancer, gossypol-induced B-cell lymphoma 2 (Bcl-2)-dependent autophagy and apoptosis through an increase in the level of the tumor protein 53 (p53) upregulated modulator of apoptosis (p53-upregulated modulator of apoptosis [Puma] and NAPDH oxidase activator [Noxa]) [9-11]. Gossypol was also found to induce autophagy in breast cancer [12], and to suppress colon cancer by downregulating cyclin D1 expression, causing G_0_/G_1_ phase arrest and activating death receptor 5 (DR5; tumor-necrosis-factor-related apoptosis-inducing ligand receptor-2, or TRAILR-2)–mediated apoptosis [13,14]. In some cases, gossypol inhibited extra-large B-cell lymphoma (Bcl-xL) in large cell lymphoma and non-Hodgkin lymphoma [15,16], and in others gossypol suppressed nuclear factor kappa-light-chain-enhancer of activated B cells (NF-κB) related signaling in the U937 leukemia cell line [17]. Studies have further indicated that gossypol inhibits lung cancer, ovarian cancer, and pancreatic cancer [18,19]. We therefore supposed that gossypol may also be a potential candidate for treating retinoblastoma.

## Methods

### Preparation of gossypol

Gossypol (Tocris Bioscience, Bristol, UK) was dissolved in dimethyl sulfoxide (DMSO) to make a 25 mM stock solution. The stock solution was diluted to concentrations of 0.5, 1, 3, 5, 10, and 20 mM in DMSO before the experiments were started, and then added (1 in 1,000) to the cell culture medium.

### Cell culture

The human retinoblastoma Y79 (HTB-18) and the human retinal pigmented epithelium (ARPE) cell lines were purchased from Bioresource Collection and Research Center (BCRC, Hsinchu, Taiwan). Cells were cultured in RPMI 1640 medium (Gibco, Life Technologies, Grand Island, NY) containing 10% fetal bovine serum (FBS) and 1% penicillin–streptomycin. The cells were incubated at 37 °C with 5% CO_2_, and the culture medium was changed every 3 days.

### Cell viability assay

Cell viability was measured with the 3-(4,5-methylthiazol-2-yl)-2,5-diphenyltetrazolium bromide (MTT) assay. Y79 cells (5×10^4^ cells/well) were cultured in 24-well plates with 1 ml RPMI 1640 medium and 0.5, 1, 3, 5, 10, or 20 μM of gossypol for 6 to 48 h. The preparations for the control group were cultured without gossypol but with the same amount of DMSO as the experimental groups. After incubation, 100 μl of MTT was added in each well, and incubated at 37 °C for another 2 h. Triton (10 μl, 10%) solution was then added into the preparations to dissolve the purple formazan from each cell. The amount of soluble formazan was measured with an enzyme-linked immunosorbent assay (ELISA) plate reader (AD200; Beckman Coulter, Brea, CA) at 550 nm absorption.

### Assay for nuclear condensation

Y79 cells were cultured in each well of a 24-well culture plate (5×10^4^ per well) with 1 ml RPMI 1640 medium, and treated with 0, 1, 10, or 20 μM of gossypol for 24 h. After incubation, cells were collected and centrifuged at 92.5 ×g for 5 min at room temperature, washed twice in phosphate-buffered saline (PBS), fixed with 4% formaldehyde at room temperature for 30 min, and washed with PBS twice again after fixation. These fixed cells were further incubated with 4,6-diamidino-2-phenylindole (DAPI; 1 μl/ml) for 30 min at room temperature, avoiding light exposure. After DAPI staining, the cells were washed with PBS twice to remove the redundant fluorescent dye, the remaining liquid was removed, and the preparation was completely resuspended in the antifading mounting medium (Dako, Produktionsvej, Denmark). The preparation was then transferred to glass slides for observation under a fluorescence microscope (DM2500; Leica, Wetzlar, Germany), and images were taken and counted using a cooled charge-coupled device (CCD) camera (CoolSNAP EZ; Roper Scientific, Martinsried, Germany).

### Annexin V-fluorescein isothiocyanate and propidium iodide staining

After treatment with different concentrations of gossypol, cells were collected under the same conditions as described earlier, except that they were stained with annexin V-fluorescein isothiocyanate (FITC) and propidium iodide (PI), instead of DAPI. Cells were washed twice with PBS and then resuspended in 500 μl of binding buffer; 5 μl of annexin V-FITC and 5 μl of PI (Annexin V-FITC Apoptosis Kit; BioVision, Milpitas, CA), and then incubated at room temperature for at least 10 min in the dark. After staining, the preparations were washed and transferred to glass slides for observation under a fluorescence microscope (DM2500; Leica) with a cool CCD camera (CoolSNAP EZ; Roper Scientific).

### Flow cytometry

Y79 cells (1×10^6^) were cultured in six-well plates with 2 ml of RPMI 1640 medium (Gibco) and 0, 1, 10, and 20 μM of gossypol for 24 h. After incubation, these cells were collected and washed in PBS, and then stained using an annexin V-FITC and PI kit (Annexin V-FITC Apoptosis Kit; Bio Vision). The preparations were transferred into the sample tube and diluted with PBS (the total sample solution contained 500 μl of binding buffer, 5 μl of annexin V-FITC, 5 μl of PI, and 490 μl PBS). For cell cycle study, the cells were stained with PI only. Data were collected from 4×10^4^ cells in the gated region of each preparation.

### Western blot analysis

Y79 cells were treated with 20 μM of gossypol for 24 h before collection. After treatment, cells were washed and resuspended in protein extraction solution (iNtRON Biotechnology, Gyeonggi-do, Korea) with 0.1% EDTA and then stored at −20 °C. Total cellular protein extract was resolved using 10 or 12% sodium dodecyl sulfate–PAGE and electrophoretically transferred to Hybond-P polyvinylidene fluoride membrane (Amersham, Buckinghamshire, UK). The blot was blocked with 5% non-fat dry milk in TBST (Tris-buffered saline [TBS] buffer, 20× liquid [AMRESCO Inc., Solon, OH]) containing 0.1% Tween-20) for 1 h at room temperature, and then incubated with primary antibodies (actin, DR5, caspase 9, caspase 8, caspase 3, cytochrome C, second mitochondria-derived activator of caspases [Smac], and p53) in blocking solution at 4 °C overnight. After incubation, the blot was washed in TBST, incubated for 1 h at room temperature, and then incubated with horseradish peroxidase (HRP)-linked secondary antibody. The binding antibodies were detected using an electrochemiluminescence (ECL) assay.

### Statistical analysis

Experimental data were analyzed by using SPSS version 18 (IBM, Armonk, NY). The Student *t*-test and one-way ANOVA with the Bonferroni multiple comparisons were used to evaluate statistical significance.

## Results

### Effective concentrations of gossypol

**Figure 1:**
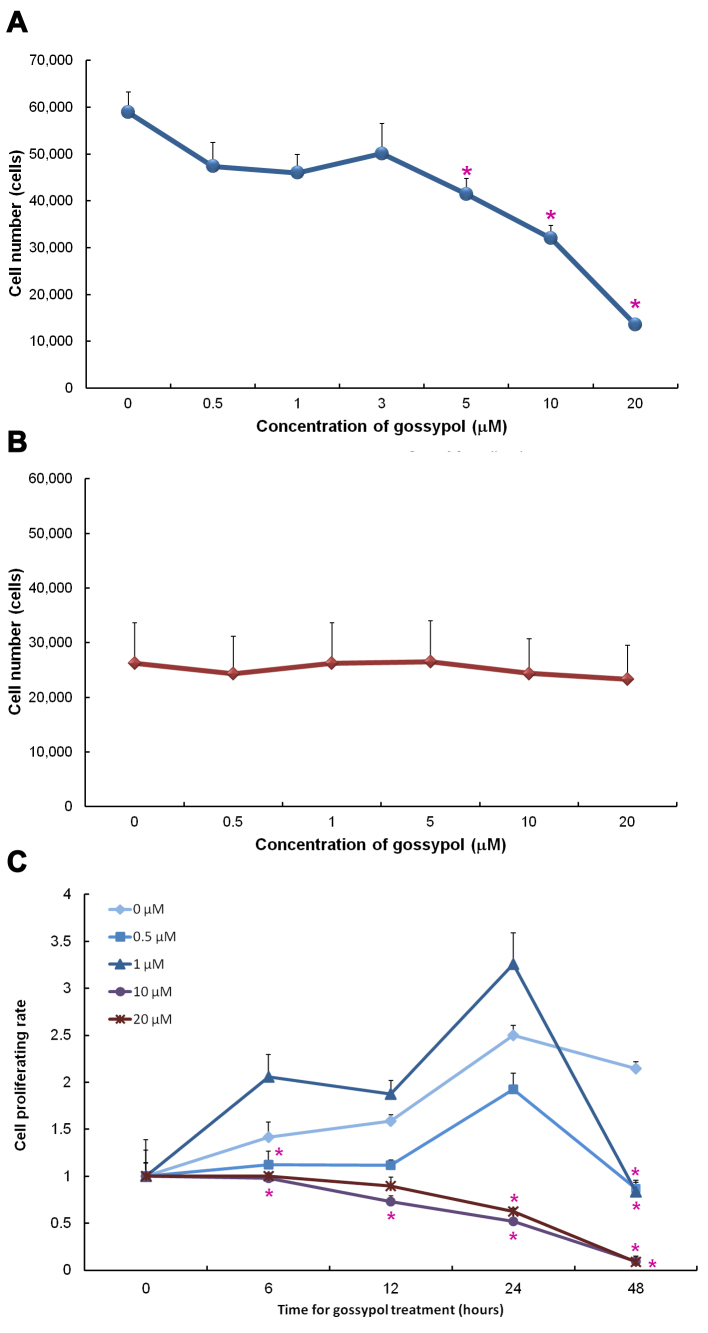
Cell viability of retinoblastoma cells detected with 3-(4,5-dimethylthiazol-2-yl)-2,5-diphenyltetrazolium bromide (MTT) assay. The effects of gossypol in different concentrations on human retinoblastoma (Y79) cells (**A**) and human RPE (ARPE) cells (**B**) are shown. Cells were cultured at different concentrations of gossypol for 24 h. Gossypol was found to decrease Y79 cell numbers at concentrations of 5, 10, and 20 μM on retinoblastoma cells but had no effect on RPE cells. Time courses of different concentrations of gossypol are shown in (**C**). Data are presented as mean±SEM. One-way ANOVA with Bonferroni multiple comparisons was used for statistical analysis. n=12–32 in (**A**), 8–9 in (**B**), and 6–12 in (**C**). * p<0.05, compared to the control group.

Considering the proven anticancer properties of gossypol, it might be supposed to mitigate human retinoblastoma as well. The present study therefore sought to determine the concentrations at which gossypol would effectively retard proliferation in the Y79 cell line. In test samples of 5, 10, and 20 μM gossypol, cell viability after 24 h of incubation was observed to be inversely proportional to the concentration of gossypol, with the ED_50_ calculated as 8.4 μm (Figure 1A). Identical dosages of gossypol were found to have no effect on APRE cells (Figure 1B).

In time-course trials, although cell viability was first seen to decrease at 6 h in the 10- and 20-μM samples, the effect remained insignificant after as long as 12 h, even at a higher concentration (Figure 1C). Accordingly, a 24-h incubation period was chosen for the subsequent investigations, which were conducted using gossypol in concentrations of 0, 1, 10, and 20 μM.

### Apoptotic studies of Y79 cells treated with gossypol

**Figure 2:**
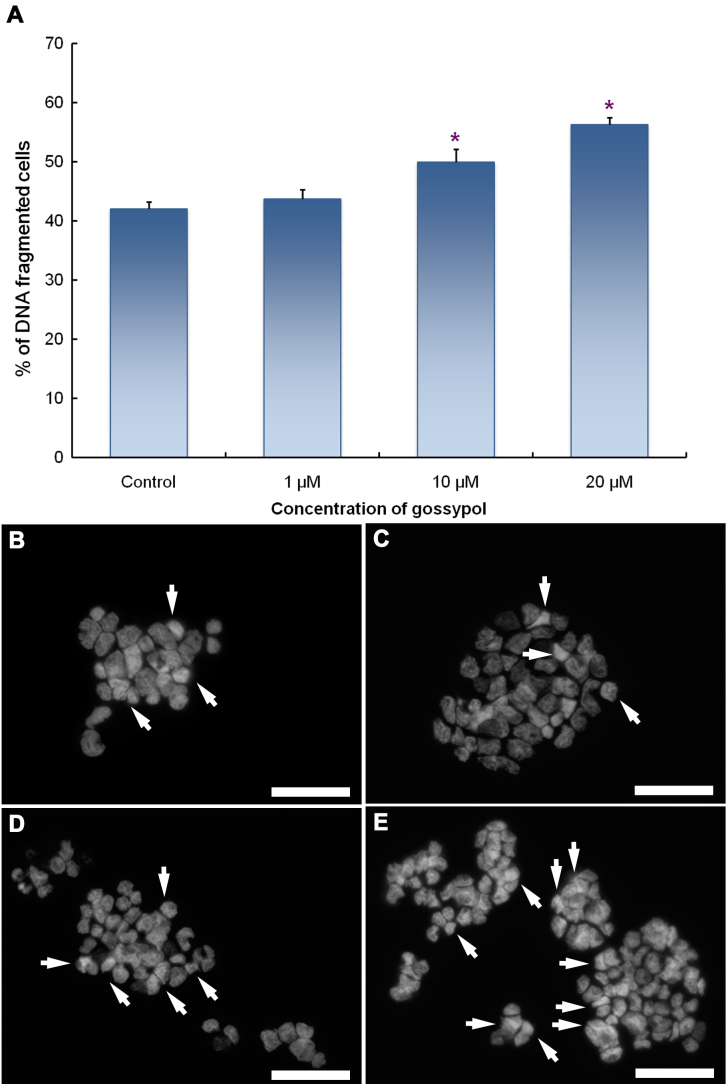
Nuclear condensation of apoptotic human retinoblastoma (Y79) cells was detected with 4,6-diamidino-2-phenylindole (DAPI) labeling. Gossypol-treated Y79 cells containing condensed nuclei were counted under a fluorescence microscope. Panel **A** shows the percentages of condensed-nucleus (apoptotic) cells. Data are represented as the mean±SEM n=14, and * p<0.05, compared to the control group. The remaining figures showing microimages show apoptotic (arrows) and normal cells in (**B**) control and in (**C**) 1 μM, (**D**) 10 μM, and (**E**) 20 μM gossypol treatments. Scale bar is 50 μm.

**Figure 3:**
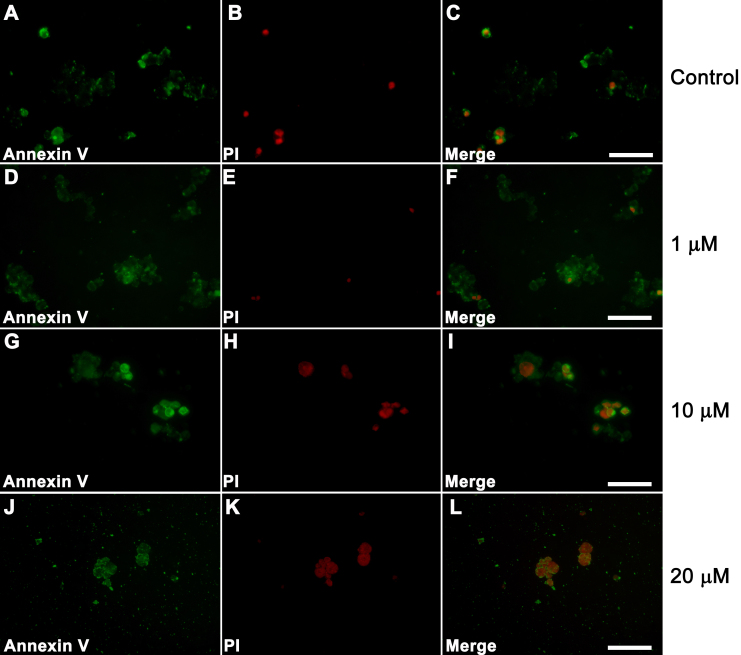
Human retinoblastoma (Y79) cells stained with annexin V and propidium iodide (PI) showing the different stages of apoptosis. **A**, **B**, and **C** are control groups. **D**, **E**, and **F** are 1 μM gossypol. **G**, **H**, and **I** are 10 μM gossypol. **J**, **K**, and **L** are 20 μM gossypol cultures. Scale bar is 50 μm.

For the apoptotic studies, Y79 cells were incubated in solutions of 0, 1, 10, or 20 μM of gossypol for 24 h. The cells were then harvested and stained with DAPI or by using an annexin V/PI apoptosis kit to observe nuclear condensation and stages of cell apoptosis. As shown in Figure 2, the rate of condensed DNA, which indicates cell apoptosis, increased in conjunction with gossypol concentrations (Figure 2A). Figure 2B shows microimages of DAPI-stained cell nuclei exposed to different concentrations of gossypol. The arrows indicate the onset of nuclear condensation, which became obvious as the concentration of gossypol increased. In addition, there was a higher incidence of cells stained with annexin V and/or PI in the samples treated with higher concentrations of gossypol than in those treated with lower or zero concentrations (Figure 3).

**Figure 4:**
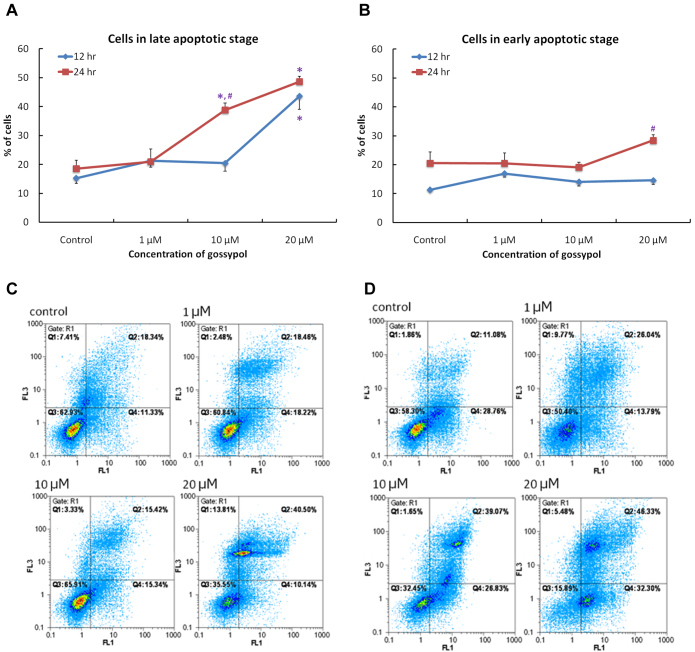
Flow cytometry demonstrated the stages of cell apoptosis in human retinoblastoma (Y79) cells at different time points and for different concentrations of gossypol. Panels **A** and **B** show the percentages of different apoptotic stages in cells incubated with different concentrations of gossypol for 12 and 24 h. Data are presented as the mean±SEM. One-way ANOVA with a Bonferroni multiple comparison was used for statistical analysis. * p<0.05, compared to the control group. **#** p<0.05, compared to the 12-h group at the same dose. Images (**C**) and (**D**) show the distribution of cells in different apoptotic stages with gossypol treatment for (**C**) 12 h and (**D**) 24 h, by collecting the annexin V signals as FL1 and propidium iodide (PI) signals as FL3. n=6 in each group. Q3 shows the normal cell population; Q4 presents the early apoptosis stage and Q2 the late apoptosis stage.

Flow cytometry was used to calculate the distribution of cells in normal, early, and late stages of apoptosis. Compared to control groups, significant increases in the proportion of cells in late apoptosis were observed in groups treated with 10 and 20 μM of gossypol for 24 h (Figure 4A). However, in preparations treated for 12 h, only the 20-μM groups showed significantly more cells in the late apoptosis stage (Figure 4A,B). Cell distributions after 12-h and 24-h treatments are shown in Figure 4C,D, where Q3 represents the normal cell population, Q4 represents cell in early apoptosis stage, and Q2 represents cells in late apoptosis stage.

**Figure 5:**
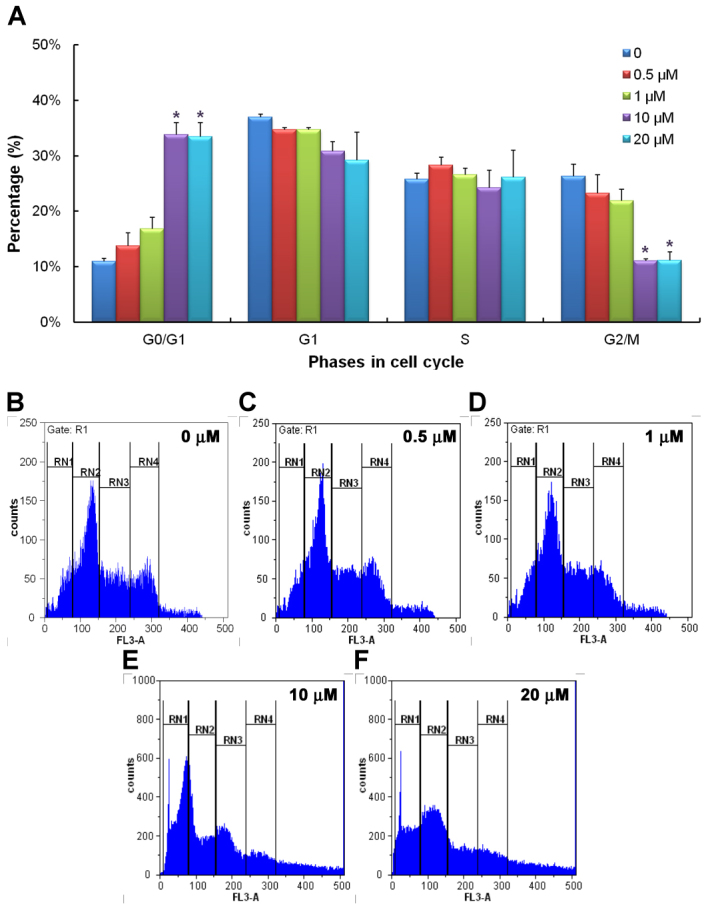
Flow cytometry demonstrated the stages of cell cycle in human retinoblastoma (Y79) cells at different concentrations of gossypol. Panel **A** shows the percentages of cells distributed in different cell cycle stages incubated with different concentrations of gossypol for 24 h. Data are presented as the mean±SEM. One-way ANOVA with a Bonferroni multiple comparison was used for statistical analysis. * p<0.05, compared to the control group. Panels **B** to **F** show the distribution of cells in different cell cycle stages in different concentrations of gossypol. n=3–4. RN1 represents the G0/G1 phase, RN2 the G1 phase, RN3 the S phase, and RN4 the G2/M phase.

The effects of gossypol on cell cycles in the Y79 cell line are shown in Figure 5. At concentrations of 10 and 20 μM, significantly more cells are in the G_0_/G_1_ phase but significantly fewer are in the G_2_/M phase than in the control groups. These results indicate that, in addition to provoking apoptosis in Y79 cells, gossypol also causes cell arrest in the cell cycles.

### Signal transduction pathway of gossypol-mediated apoptosis of Y79 cells

**Figure 6:**
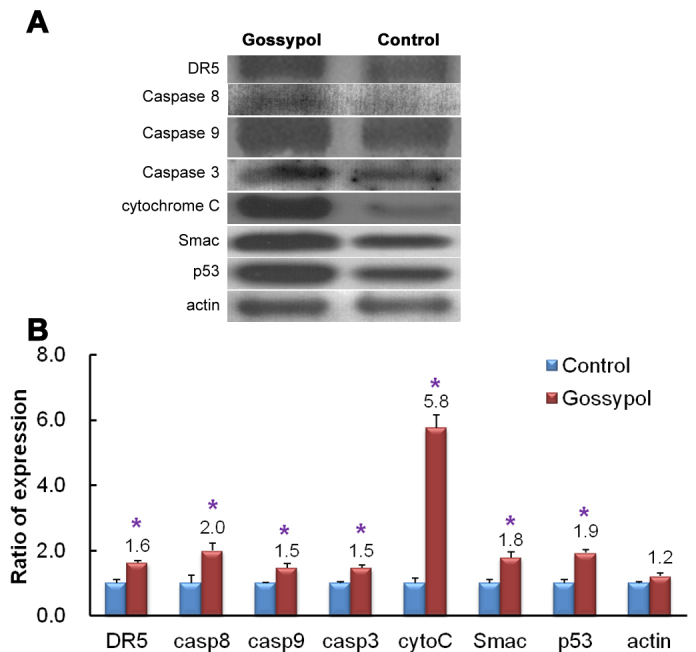
Protein expressions of extracts from control and 20 μM gossypol-treated human retinoblastoma (Y79) cell cultures were detected with western blotting. Panel **A** shows the target signal transduction proteins from the control and gossypol-treated groups. Panel **B** shows the ratio of increased expression in these proteins. The results indicate that the expressions of cytochrome C, caspase 8, caspase 9, caspase 3, DR5, p53, and Smac were upregulated by gossypol treatment. The Student *t*-test was used for statistical analysis. * p<0.05, compared to the control group, and n=3 in each group in (**B**).

Considering the potential of gossypol in treating human retinoblastoma, it is important to investigate the signaling transduction pathway via which gossypol causes apoptosis in Y79 cells. Comparison of the signal intensity of various apoptosis-related antibodies shows that the expressions of apoptotic proteins such as DR5, p53, Smac, caspase 8, caspase 9, and caspase 3 were upregulated 1.5 to twofold in the 20 μM gossypol-treated groups, compared to the control group (Figure 6). The expression of cytochrome C was upregulated by up to 5.8 fold (Figure 6B). Two signaling pathways may therefore be involved: (1) a TRAILR-mediated (death receptor 5) pathway in which the upstream caspase family members are upregulated, causing Smac and cytochrome C to be released from the mitochondria; and (2) DNA degradation causing p53 upregulation and then cell cycle arrest.

## Discussion

The present study found that the polyphenol molecule gossypol induced apoptosis in Y79 cells at concentrations of 5, 10, and 20 μM. This effect was not seen in non-cancer retinal pigmented epithelial cells. The proportion of nuclear condensed cells and the population of apoptotic cells were also found to increase significantly as the dose of gossypol increased. Cells treated with gossypol were found more likely to enter late apoptosis and to undergo cell arrest than cells in the control group.

**Figure 7:**
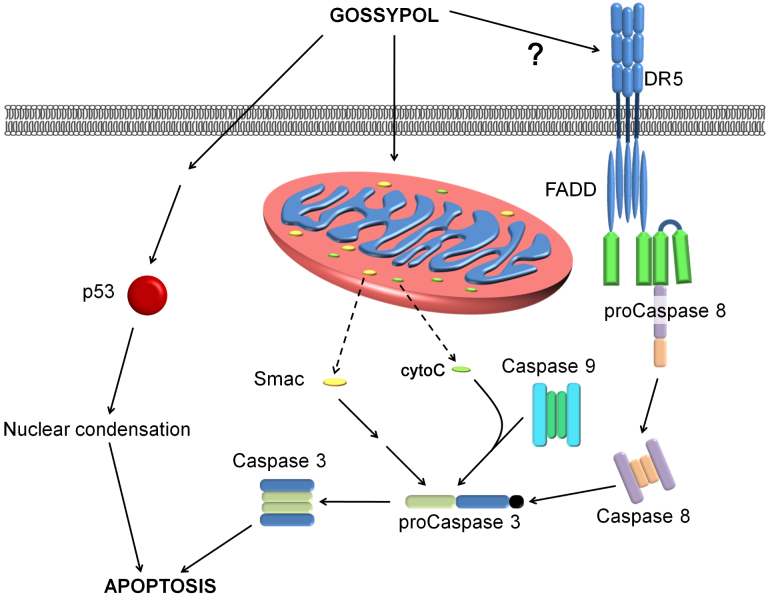
Possible mechanisms of gossypol-induced cell apoptosis in human retinoblastoma (Y79) cells. Gossypol may use several pathways leading to the death of Y79 cells, including activating death receptors (DR5), caspase 8, and caspase 3; upregulating mitochondria permeability, thereby causing the release of cytochrome C; and activating the Smac pathway. In addition, p53 was found upregulated after gossypol treatment, suggesting that DNA degradation is another effect of gossypol. Solid lines with arrows in the figure represent simulative effects, and the dotted lines represent translocation.

A gossypol dose of 20 μM was used to study the signal transduction pathway by which gossypol has its antiproliferative effect on Y79 cells. The p53, Smac, and caspase family pathways are all involved in the induction of cell apoptosis (Figure 7). The results of apoptosis protein studies through western blot analysis revealed that gossypol upregulates DR5 and activates caspase 8 to cleave procaspase 3 into activated caspase 3, thus inducing apoptosis of the cell. Gossypol also initiates activation of caspase 9, leading to the release of Smac and cytochrome C from the mitochondria. Because gossypol is lipid-soluble, it might have the ability to pass directly through the cell membrane and enhance mitochondrial or endoplasmic reticulum stress.

Earlier studies have shown gossypol to be a small-molecule non-peptide BH3-domain-binding inhibitor that blocks Bcl-2, Bcl-xL, and myeloid cell leukemia (Mcl-1) [20-22]. Gossypol also blocks the Bcl-2-homology-3 (BH3)-binding site of many antiapoptotic Bcl-2 family members that bind with other proapoptosis proteins such as Bcl2 interacting mediator of cell death (Bim), BH3 interacting domain death agonist (Bid), Bcl2-associated agonist of cell death (Bad), Puma, and Noxa, thus destabilizing the mitochondria membrane and effectuating apoptosis. Some research has shown gossypol to have a downregulation effect on Bcl-2, Bcl-xL, and Mcl-1. However, our results indicate no significant change in the expression of Mcl-1, Bcl-2, and Bcl-xL after gossypol treatment. Changes in these antiapoptotic proteins have been postulated by other researchers [10,16]. The mechanism of the upregulation of Mcl-1 is thought to be caspase-independent [10], and there is evidence that gossypol directly interferes with antiapoptotic protein function rather than expression level [16]. Gossypol might also block the BH3-binding site of endoplasmic reticulum-associated proteins such as Beclin 1 and induce autophagy in cells. We found no evidence showing a similar effect of gossypol on Y79 cells.

The upregulation of mitochondria-related apoptotic proteins observed in the present study suggests that gossypol molecules induce mitochondrial stress, thereby increasing mitochondrial membrane permeability and causing Smac and cytochrome C to be released through the membrane to the cytoplasm. This might lead to procaspase 3 cleavage and subsequent cell apoptosis. Some researchers have suggested that this protein release mechanism shares the same pathway as Bak- and Bax-dependent cytochrome C release [23]. Because of the present results showing that gossypol initiates upregulation of p53, we supposed that gossypol may also mediate nuclear degradation directly via this pathway.

In our preliminary protein array screening assay, the expression of insulin-like growth factor-binding protein 6 (IGFBP-6) increased more than twofold in the gossypol-treated preparations. Other studies have indicated that Y79 occasionally uses autocrine signaling through insulin-like growth factor (IGF) types I and II to stimulate cell growth [24]. IGFBP-6 has the highest affinity to IGF II of all IGFBP members, which could mean that in gossypol-treated preparations, IGF II could compete with insulin-like growth factor receptors (IGFRs) and prevent IGF-mediated signaling activation [25]. IGFBP-6 has an antiproliferative and apoptotic effect on many types of IGF II-dependent tumors [26], and our results suggest a similar effect on this pathway in Y79 cells.

Gossypol has been shown to have antiangiogenesic [27,28] and antimetastatic [29,30] effects in many other cancers. This study concludes that gossypol induces cell apoptosis by inducing mitochondrial stress, DNA damage, and cell arrest in retinoblastoma cells, and that gossypol does not damage non-cancer retinal pigmented epithelial cells. Gossypol may therefore have a clinical application in anticancer therapy; however, further investigations with in vivo studies are required.
